# Bedside Coagulation Tests in Diagnosing Venom-Induced Consumption Coagulopathy in Snakebite

**DOI:** 10.3390/toxins12090583

**Published:** 2020-09-10

**Authors:** Supun Wedasingha, Geoffrey Isbister, Anjana Silva

**Affiliations:** 1 Faculty of Medicine and Allied Sciences, Rajarata University of Sri Lanka, Saliyapura 50008, Sri Lanka; swnadeeja@gmail.com; 2 Clinical Toxicology Research Group, University of Newcastle, Newcastle, NSW 2308, Australia; geoff.isbister@gmail.com; 3 Monash Venom Group, Department of Pharmacology, Faculty of Medicine, Nursing and Health Sciences, Monash University, Melbourne, VIC 3800, Australia

**Keywords:** clotting test, bedside, snakebite, envenoming, venom-induced consumption coagulopathy

## Abstract

Venom-induced consumption coagulopathy is the most important systemic effect of snake envenoming. Coagulation tests are helpful to accurately and promptly diagnose venom-induced consumption coagulopathy and administer antivenom, which is the only specific treatment available. However, bedside clotting tests play a major role in diagnosing coagulopathy in low-income settings, where the majority of snakebites occur. We conducted a literature search in MEDLINE^®^ from 1946 to 30 November 2019, looking for research articles describing clinical studies on bedside coagulation tests in snakebite patients. Out of 442 articles identified, 147 articles describing bedside clotting assays were included in the review. Three main bedside clotting tests were identified, namely the Lee–White clotting test, 20-min whole blood clotting time and venous clotting time. Although the original Lee–White clotting test has never been validated for snake envenoming, a recently validated version has been used in some South American countries. The 20-min whole blood clotting time test is the most commonly used test in a wide range of settings and for taxonomically diverse snake species. Venous clotting time is almost exclusively used in Thailand. Many validation studies have methodological limitations, including small sample size, lack of case-authentication, the inclusion of a heterogeneous mix of snakebites and inappropriate uses of gold standard tests. The observation times for bedside clotting tests were arbitrary, without proper scientific justification. Future research needs to focus on improving the existing 20-min whole blood clotting test, and also on looking for alternative bedside coagulation tests which are cheap, reliable and quicker.

## 1. Introduction

Snakebite is a significant public health issue that mainly affects poor farming communities in the rural tropics and subtropics [1,2]. Since June 2017, it has been recognized by the World Health Organization as a category A—Neglected Tropical Disease [3]. Snakebites cause significant mortality and morbidity, particularly in South Asia, Southeast Asia, Sub-Saharan Africa, and Central and South America. Literature-based estimates suggest that there are 0.4 to 1.8 million envenomings and 20,000 to 90,000 deaths due to snakebite globally each year [1]. Even in the worst affected regions, snakebite mainly impacts the poorest communities [4]. Snakebite results in both acute and chronic effects of envenoming. Acute envenoming can cause local swelling and necrosis at the bite site, venom-induced consumption coagulopathy (VICC), neurotoxicity, acute kidney injury, myotoxicity and cardiovascular effects [5]. Chronic effects include both medical and psychological illness [6]. Overall, this causes a significant socioeconomic burden to the effected communities [7].

## 2. Venom Induced Consumption Coagulopathy

Venom-induced consumption coagulopathy (VICC) is the most common and most important systemic effect of snake envenoming worldwide [8]. True and pit vipers (Family: Viperidae) and Australasian elapids (Family: Elapidae) are known to cause VICC in envenomed humans [8,9]. VICC is the broad term given to a coagulopathy resulting from consumption of clotting factors due to procoagulant toxins present in a snake venom [9]. In the past, VICC was incorrectly referred to as disseminated intravascular coagulation, because of the elevated D-dimer, prolonged prothrombin time and low fibrinogen levels that occur with VICC. However, VICC differs from disseminated intravascular coagulation due to the absence of systemic microthrombi, the absence of end-organ failure, the rapid onset and resolution, the different pathogenesis and the lower mortality [10].

Although the term ‘haemotoxins’ is often used to describe the toxins that cause VICC, it is a broad term that can be used to refer to any haemostatically active venom component, including toxins acting on coagulation and fibrinolysis, toxins which disrupt the vascular endothelium and toxins that cause platelet dysfunction. Toxins that act on coagulation factors are classified as procoagulant toxins, anticoagulant toxins and toxins that affect fibrinolysis [11]. Procoagulant toxins cause VICC by activating the clotting pathway. They cause rapid clot formation in vitro, but in vivo they lead to the rapid consumption of clotting factors and coagulopathy, with the consequent risk of bleeding [8]. They are classified according to the part of the clotting pathway they act on [9]. These include factor V and factor X activators in Russell’s viper venom [12], prothrombin activators in carpet viper venom (Genus: *Echis*) and Australasian elapid venoms [13], and thrombin-like enzymes in pit viper venoms [14]. A detailed account of the snakes and their different toxins causing VICC in humans has been provided by Berling and Isbister [8]. The activation of a clotting factor by procoagulant toxins leads to subsequent activation of other clotting factors downstream in the cascade, which get consumed in the process. The final step in the pathway is the conversion of fibrinogen to fibrin, which results in low levels (hypofibrinogenaemia) or the absence (afibrinogenaemia) of circulating fibrinogen. This and low levels of other factors (e.g., factors V and VIII) result in a coagulopathy—hence the term consumption coagulopathy [9].

Haemorrhagins are proteolytic enzymes, such as metalloproteinases, which damage the basement membrane in the blood vessel wall and in turn increase the risk of bleeding associated with consumption coagulopathy. The metalloproteinase prothrombin activating toxin ecarin in *Echis* species can also act as a haemorrhagin in addition to activating the clotting cascade. Collectively, the above toxins lead to the more severe bleeding observed in *Echis* species compared to Australian elapid envenoming [8], in the latter of which there are no haemorrhagins.

Antivenom is regarded as the only specific treatment available for VICC [15]. Antivenom is believed to bind to toxins in the blood and prevent the action of toxins, or to increase the elimination of toxins from the body [9,16]. Administration of antivenom has been shown to improve the recovery from VICC in envenoming by *Echis* species [9,17]. However, once the pro-coagulant toxins activate the clotting cascade, consuming clotting factors, the synthesis of new clotting factors is required for the coagulopathy to recover. Therefore, it is important to administer antivenom as early as possible before VICC is well established [9].

## 3. Laboratory Diagnosis of VICC

Coagulation tests are important in diagnosing and monitoring VICC in snakebite patients, and determining promptly and accurately which patients should receive antivenom [18]. The prothrombin time (PT)/International normalized ratio (INR) is considered to be the most useful diagnostic test in VICC. The activated partial thromboplastin time (aPTT) is also abnormal in VICC, but is particularly helpful in diagnosing anticoagulant coagulopathy, in which the aPTT will be elevated and the INR normal. The D-dimer measures the production of cross-linked fibrin degradation products, and is the best way to distinguish consumptive coagulopathy from other types of coagulopathies. It is markedly elevated in VICC because of the uncontrolled activation of the clotting pathway, leading to the rapid production and consumption of fibrinogen. Fibrinogen is the only easy and clinically available factor-level measurement and will be low or absent in VICC. However, it is less helpful in monitoring the recovery of VICC because it is the slowest clotting factor to recover [8]. Specific factor assays help to determine the type of procoagulant toxin responsible; for example, in VICC due to Russell’s viper, levels of factor V, factor VIII and factor X are low [12], compared to factor V and VIII levels in Australasian elapid VICC.

Most coagulation studies are not freely accessible in low-income countries, where the majority of snakebites occur. Commercial point of care coagulation studies that have become available for the community monitoring of anticoagulant therapy are also problematic for snake envenoming [19]. Therefore, several simple bedside clotting tests are used more commonly around the world. These tests vary in their methods and times of observation for clot formation [18,20].

## 4. Types of Bedside Coagulation Tests for VICC

### 4.1. Lee–White Clotting Test

In 1913, Lee and White demonstrated a simple clinical method to determine the coagulation time of blood—the Lee–White clotting time. It was originally developed to diagnose various haematological disorders and to monitor anticoagulant therapy. Subsequently, this test has been modified to detect VICC in snake envenoming. The method is as follows: One millilitre of blood is obtained from an arm vein with a needle (preferable platinum) and a small all-glass syringe, which has been sterilized with normal salt solution. The time of blood withdrawal is noted as accurately as possible. The syringe is then emptied into a small glass tube 8 mm in diameter, which has been rinsed in normal salt solution. Every 30 s the tube is inverted and the time at which blood no longer flows from its position when inverted is taken as the endpoint (Table 1) [21].

#### 4.1.1. Validation Studies of the Lee–White Clotting Test

The search did not identify a study that has evaluated, validated or used the original Lee–White method in the diagnosis of VICC. However, a slightly modified Lee–White clotting time has been used in diagnosing VICC in *Bothrops atrox*-envenomed patients from Brazil [22,23]. This modified test is performed by placing 1 mL of venous blood in a glass tube and leaving it undisturbed for 5 min. Following this, the tube is gently tilted every minute and the clotting time is taken when clot formation is observed. Although it is not clear how it was determined, the upper limit of time that is normal for this modified Lee–White clotting test has been defined as 9 min [23]. In *Bothrops atrox*-envenomed patients, the modified Lee–White clotting method was shown to have a sensitivity of 78% and a specificity of 40.7% compared to fibrinogen concentrations (>200 mg/dL was considered to be normal), when performed by laboratory technicians. During this study, it was noted that mild VICC may be present even with a negative modified Lee–White clotting time result [23].

The search found no other clinical studies that have used the Lee–White clotting test to diagnose VICC.

#### 4.1.2. Use of Lee–White Clotting Test in Snakebite Clinical Studies

The search did not find any studies that used the original Lee–White clotting test to diagnose VICC. However, a clinical study of VICC in *Echis carinatus* bites conducted in Northern Nigeria used a clotting test similar to the Lee–White clotting test [24]. This test was performed by drawing 3 mL of blood into a standard 5 mL glass syringe. The syringe was then kept horizontal for 5 min, and then moved gently at intervals, looking for evidence of clotting. An arbitrary maximum normal clotting time was defined as 10 min for this study [24]. The study presented no data on the validly of this test.

### 4.2. 20-Min Whole Blood Clotting Test and Its Variants

As a modification of the Lee–White clotting test, Warrell et al. [25] introduced a much simpler 20-min whole blood clotting test (WBCT20), which is now widely used as a bedside clotting test for diagnosing VICC. The WBCT20 was originally described as placing ‘a few milliliters of freshly sampled venous blood’ into a new, clean, dry glass vessel (tube or bottle), and leaving it undisturbed for 20 min at the ambient temperature. The vessel is tipped once at 20 min, and if the blood has not clotted and runs out, this indicates the presence of VICC and a positive test [24]. However, the basis for the 20-min time period and the validity of the test was not described in the original study of the WBCT20. The World Health Organization has recommended the WBCT20 to be used as a bedside test to diagnose VICC in low-income health care settings, so as to determine if patients require antivenom [5]. The WBCT20 has been modified in various studies, such as a large scale clinical trial held in Cameroon to assess the clinical safety and efficacy of a polyvalent antivenom, in which the observation time of the test was extended to 30 min [26]. The duration of the WBCT20, including time periods of 30 min [27], 10 min [28] and 11 min [29], has also been reported in the literature, again with little justification or evidence to support the different durations of the test. Another modification is to simply report the whole blood clotting time. For this, the tube is left undisturbed for 7 min and then tilted every minute to record the time until a clot forms [30].

The search found numerous clinical studies from a range of different settings that have used a WBCT, including from Australia [30], India [31], Sri Lanka [32], Thailand [33,34], Myanmar [35,36,37,38], Nigeria [25], Brazil [39] and Papua New Guinea [40,41]. Further, the WBCT has been used to detect VICC caused by taxonomically diverse snakes, including true vipers such as Russell’s viper (*Daboia russelii*) [12,31,34,35,36,37,38,42,43,44,45], saw-scaled viper (*Echis carinatus*) [32], pit-vipers such as *Bothrops jararaca* [39,46], South-Asian hump nosed pit vipers (*Hypnale hypnale*, *Hypnale zara*, *Hypnale nepa*) [14,28,47], Malayan pit viper (*Calloselasma rhodostoma*) [48] and green pit vipers (*Trimeresurus albolabris* and *Trimeresurus macrops*) [49,50], as well as Australasian elapids such as Western brown snake (*Pseudonaja nuchalis*) [30], mulga snake (*Pseudechis autralis*) [30], Papuan taipan (*Oxyuranus scutellatus canni*) [40,41], Papuan black snake (*Pseudechis papuanus*) [51], some colubrids such as *Philodryas olfersii* [52] and *Philodryas patagoniensis*, and lamprophiids such as Mock viper (*Tomodon dorsatus*) [53].

#### 4.2.1. Validation Studies of the 20-Min Whole Blood Clotting Test

Several studies have attempted to validate the WBCT20 for detection of VICC caused by different snakes. In 1994, Sano-Martins conducted an observational study of patients envenomed by Bothrops that showed a good agreement between fibrinogen concentration and WBCT20, both on admission and after antivenom therapy [54]. In a cohort study of 70 suspected snakebites conducted in tropical northern Australia from 1999 to 2000, a whole blood clotting test (termed “whole blood clotting time”) was used, and whole blood clotting tests with cut-offs of both 10-min (WBCT10) and 20-min were used; WBCT10 to diagnose envenoming and WBCT20 to diagnose VICC. The WBCT20 had a sensitivity and specificity of 100%, whereas WBCT10 had a sensitivity of 100% and a specificity of 60%. The good performance of the WBCT20 and WBCT10 was most likely due to the very small number of cases with actual envenoming and VICC included in the study [30].

In an observational study done in Ghana, syringes (material unspecified) and uncleaned empty ceftriaxone bottles were compared with test tubes for conducting WBCT20 in 46 sequential patients with possible snakebites [55]. Both the syringes and ceftriaxone bottles appeared to be in agreement with the test tubes in diagnosing VICC, with the syringe method having a sensitivity of 88.9% and specificity of 82.4%, and the bottle method having a sensitivity of 83.3% and specificity of 90.0%. A comparison of the areas under the receiver operator characteristics curves showed that both these tests did not differ much in their overall discrimination compared to the glass tubes. However, it is unlikely that the containers were kept undisturbed during the observation period, as the study had also described the times taken for clot formation [55].

In another observational study involving 140 cases of definite Russell’s viper bite patients from Sri Lanka, the WBCT20 was found to have a sensitivity of 40% and specificity of 100%. WBCT20 was done according to protocol by the treating team, but without proper standardization. Laboratory coagulation assays were done on frozen samples, which were not always collected at the same time as the WBCT20. This study showed that WBCT20 was not sensitive enough to detect even severe degrees of coagulopathy, because the proportion of positive WBCT20 only slightly increased with severe coagulopathy based on an INR. Despite the poor sensitivity of WBCT20, all patients received antivenom. However, one-third of patients had a delayed administration of antivenom, and antivenom was given in almost half of the envenomed patients without a prior positive WBCT20 [18]. A subsequent clinical study was undertaken in the same setting on authenticated snakebites, with the WBCT20 performed by trained research assistants using new clean glass tubes with standard dimensions. This standardized approach improved the sensitivity to 82% with a specificity of 98%, compared to the gold standard (INR 1.4 as the upper normal limit) [20]. Coagulation assays were performed immediately after the blood collection with fresh samples at the same time as WBCT20. Despite the better standardization of WBCT20, it still missed around one-fifth of patients who should have received antivenom [20].

A clinical study done in India showed that WBCT20 had a sensitivity of 50% and specificity of 89.13%, using an INR > 1.5 as the upper normal limit. However, case-authentication and the exact method of WBCT20 was not described in this study [56]. In another study from India of 60 snakebite patients, the WBCT20 had a sensitivity of 94% and specificity of 76%, in which the presence of clinically detectable envenoming (such as local features, bleeding and neurotoxicity) was taken as the gold standard. However, this study focused on the detection of envenoming rather than VICC [57].

#### 4.2.2. Use of WBCT20 in Clinical Studies

The WBCT20 has been used as the sole assessment method of VICC in numerous clinical studies, including non-randomized controlled clinical trials [58], randomized comparative trials [59,60,61,62,63,64], non-randomized comparative trials [29,65], observational studies [27,31,32,35,37,41,43,47,50,52,53,66,67,68,69,70,71,72,73,74,75,76] and case series [77,78,79,80].

The WBCT20 has also been used to diagnose VICC in combination with coagulation tests, such as PT/INR, aPTT, fibrinogen and fibrin degradation products, in a number of clinical studies, including randomized controlled trials [81,82], randomized comparative trials [83,84,85,86,87,88,89], observational studies [14,25,33,36,45,48,49,51,90,91,92,93,94,95,96,97,98,99,100,101] and case series [102,103,104].

### 4.3. Venous Clotting Time

The venous clotting time (VCT) is a widely used bedside test in Thailand for snakebite management. The test is performed by drawing 3 mL of blood from a vein and placing 1 mL in 3 glass tubes in sequence at room temperature. The first tube is labelled as 3, the next as 2, and the last one as 1. A timer is started when the blood touches the first glass tube. They are then kept still for 5 min. After 5 min, tube 1 is tilted first about 45 degrees every 30 s to 1 min until a clot is seen, then tube 2 the same way as tube 1, and finally tube 3. The time from starting the timer until the blood in tube 3 turns entirely into a clot is taken as the VCT [105]. This test is thought to indicate mild coagulopathy when it exceeds 20 min, and severe coagulopathy when it exceeds 30 min. The VCT is almost exclusively used in Thailand to diagnose VICC due to pit vipers, green pit vipers (*Trimeresurus albolabris* or *T. macrops*) [106,107] and Malayan pit viper (*Calloselasma rhodostoma*) [108].

#### 4.3.1. Validation Studies of the Venous Clotting Time

In a prospective observational study in Thailand, the 15-min VCT, 20-min VCT, 30-min VCT and WBCT20 yielded sensitivities of 71.4%, 57.1%, 57.1% and 85.7% respectively, and specificities of 71.1%, 84.4%, 94.4% and 95.8% respectively, in comparison to the gold standard, fibrinogen level (normal lower limit of 1 g/L). The sensitivity of VCT in detecting hypofibrinogenaemia was low when compared to WBCT20. The VCT was also more complicated and subject to analytical errors, and some of the VCTs were performed by physicians instead of laboratory personnel. The WBCT20 not being done in all patients may also have influenced the final results of this study [109].

#### 4.3.2. Use of Venous Clotting Time in Clinical Studies

Our literature search yielded four studies which used the VCT in assessing VICC, including randomized controlled trials [110,111] and observational studies [106,112].

## 5. Novel Methods to Diagnose VICC

An aPTT-based clot waveform analysis, which is an optic absorbance assay used as a global clotting test, was successfully used in a group of Russell’s viper bite victims in diagnosing VICC. It could detect abnormalities in coagulopathy before conventional clotting tests could [113]. Compared to traditional coagulation tests, thromboelastography (TEG) has few advantages. It is less time-consuming and it can provide certain information that the PT and aPTT cannot, including fibrin strength and pseudo-procoagulant effect. However, TEG is too expensive to be used in low-income settings in which most snakebites occur. There have been no randomized controlled studies undertaken to assess the utility of TEG in patients with VICC [114].

Another clinical study demonstrated that a simple phospholipase A_2_ assay can be used as a potential bedside test to identify systemic envenomation in snakebite patients, including coagulopathy [115].

## 6. Discussion

Our literature search identified three main types of bedside coagulation tests that have been used to assess VICC, namely the Lee–White clotting test, the WBCT20 and venous clotting time. The original Lee–White clotting test has never been validated for snake envenoming, and only a modified version has been recently validated. The WBCT20 is the most commonly used test in a wide range of settings, and has been validated in some settings for detecting VICC in envenoming. The venous clotting time is validated and used almost exclusively in Thailand to detect VICC in green pit viper envenoming. However, the test is complex compared to the WBCT20.

The WBCT20 is a cheap and simple test that can easily be performed at the bedside even in rural health care settings. Currently, there is no consensus on tube type, the diameter of the tube, the volume of blood to be collected and other factors that could influence the performance of the test. Various studies have employed different observation times for WBCT. It has been included in the guidelines of the World Health Organization, but there is no standardization or instruction as to how to do the test. In a recent validation study of the WBCT20, a standardized approach was developed: 1 mL of venous blood was placed in 5 mL glass tubes with a 10 mm internal diameter [20]. However, even a 20-min observation time may be too long for some cases of severe VICC, resulting in a delay in the administration of antivenom.

Many of the validation studies had methodological issues, such as a small sample size, a lack of case-authentication, the inclusion of a heterogeneous mix of snakebites and the use of inappropriate gold standard tests such as an INR or fibrinogen levels. Almost all the validation studies of bedside clotting tests have not provided justifications for their choice of upper normal INR and lower normal fibrinogen levels in their respective definitions of VICC. In a snakebite victim, the severity of VICC must be assessed together with the risk of bleeding in order to achieve a clinically meaningful judgement on the necessity of antivenom. VICC with an INR 3 may be different in eastern brown snake (*Psuedonaja textillis*) envenoming compared to Russell’s viper (*Daboia siamensis*/*D. russelii*) envenoming, as the latter snake causes spontaneous bleeding manifestations more commonly than the former, due to the presence of haemorrhagic toxins in the venom [36,38,116]. Because of the species differences in the mechanisms of VICC and the impact of haemorrhagic toxins, defining the upper normal limit of INR and lower normal limit of fibrinogen in a clinically meaningful way is important when using them as gold standard tests for VICC, and in validating bedside clotting tests.

In the original descriptions and subsequent validation studies, the observation times for bedside clotting tests have not been scientifically justified within the context of VICC, and appear to be arbitrary values. The duration of VICC and the severity of the outcomes of VICC depend on the type of pro-coagulant toxins involved. A mild degree of VICC occurs when there is only fibrinogen consumption, as in many pit viper bites, compared to more severe VICC with multiple factor consumption, as in the case of envenoming by Australian elapids and Russell’s vipers [9,10]. Therefore, it could be argued that using a fixed reference point of time for bedside clotting assays in diagnosing VICC for different snake species may not always be appropriate. Hence, case-authentication and accurate snake identification in such validation studies is important. Further, the effect of the bite-to-test time gap on the performance of the test has not been addressed in the original descriptions and validation studies of the bedside tests, apart from in one validation study of WBCT20 [20]. If the bedside test is less sensitive to mild VICC and highly sensitive to complete VICC, a patient with mild VICC on admission to the hospital might be missed by the test. The same patient may have a positive test later if more severe VICC develops, and so miss the chance for early detection of VICC and early antivenom administration. Therefore, the bite-to-test time should be taken into account when assessing the performance of the test.

Many clinical studies have not clearly stated or have incorrectly named the type of bedside clotting test used. There were three clinical trials of antivenom that have cited the Lee–White method as their method of assessment of VICC. However, the scanty details given in the methodology suggest that these trials had used the WBCT20 method. The terms ‘Lee–White clotting test’ and ‘Warrell’s WBCT20’ have been used interchangeably [59,85,86]. Vague terms such as ‘clotting time’ and ‘coagulation time’ are sometimes used in clinical trials [117,118] and observational studies [119,120,121,122], which further confuses the value and use of these tests.

Because no bedside clotting test is perfect, clinicians should consider the full clinical picture of the individual snakebite patient in addition to the bedside clotting test. Future research needs to focus on validating the existing WBCT against a gold standard coagulation test combined with envenomation criteria, and also how it can be adapted to be a more reliable, flexible and practical bedside tool for different species of snakes. The use of a capillary blood clotting test for the detection of VICC has been underexplored, but appears to be promising [123]. Future research needs to focus not only on improving the accuracy of bedside clotting tests, but also on investigating novel rapid tests to diagnose VICC [115]. It is high time to look for alternative bedside coagulation tests that are cheap, reliable, quick and practical enough to be performed in rural health care settings, where the majority of snakebites occur.

## 7. Methods

**Figure 1:**
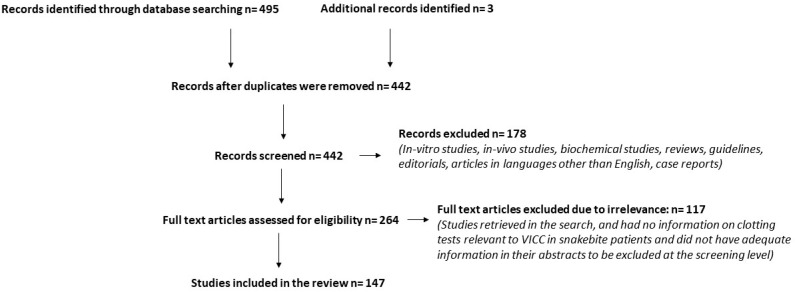
Selection of the studies for the review.

A literature search was carried out in MEDLINE^®^ from 1946 to 30 November 2019 looking for research articles that describe clinical studies of bedside coagulation tests in snakebite patients. The following search terms were used: “whole blood clotting time”, “whole blood coagulation time”, “whole blood clotting time 20”, “bedside clotting time”, “bedside clotting test”, “Lee–White clotting time”, “venous clotting time”, “clotting time”, “snakebite”, “snake envenomation”, “snake envenoming”, “venom-induced consumption coagulopathy”, “VICC”, “coagulopathy” and “bleeding”. Case reports were excluded. This search yielded 495 articles. Reference lists of retrieved articles were searched for additional articles as well. After removing duplicates, a total number of 442 articles was found (Figure 1). We then excluded in vitro studies, in vivo studies, biochemical studies, reviews, guidelines, editorials and articles in languages other than English. This resulted in 264 articles. The full text of each of the 264 articles was reviewed and a further 117 studies were excluded. The review included 147 studies.

## Figures and Tables

**Table 1:** Comparison of bedside clotting tests used in the detection of venom-induced consumption coagulopathy.

	Lee–White Clotting Method	Modified Lee White Clotting Time	WBCT20 Method	Venous Clotting Time
Type of blood	venous	venous	venous	venous
Volume of blood	1 cc of blood	1 cc of blood	‘a few milliliters’	1 cc of blood in each tube
Vessel	Glass tube	Glass tube	Glass tube or bottle	Glass tube
Specifications of the vessel	8 mm in diameter			
Method	Blood is taken from an arm vein using a small all glass syringe which has been sterilized with a normal salt solution, preferably with a platinum needle Syringe is then emptied into a tube which has also been rinsed with a normal salt solution Every 30 s tube is rotated endwise	Venous blood is placed in a glass tube and the tube is left undisturbed for 5 min. Then the tube is gently tipped every following minute	Few milliliters of venous blood is placed in a new, clean, dry, glass vessel and left undisturbed for twenty minutes at room temperature Vessel is tipped exactly at 20 min	1 cc of blood is placed in each tube in sequence at room temperature First tube is labeled as 3, next one as 2 and the last one as 1 A timer is started when blood touches the first tube Three tubes are kept still for five minutes After 5 min, tube 1 is tilted first about 45 degrees every 30 s to 1 min until a clot is seen, then tube 2 the same way and next tube 3
Result reading point	Point at which blood no longer flows from its position when inverted		20 min	Time from starting the timer until blood in tube 3 turns to a clot
